# Decreased coronary artery distensibility measured using 3T MRI is related to systemic inflammation in patients with coronary artery disease

**DOI:** 10.1186/1532-429X-17-S1-P400

**Published:** 2015-02-03

**Authors:** Lia Petrose, micaela iantorno, Sahar Soleimanifard, Sebastian Kelle, Gary Gerstenblith, Robert Weiss, Allison Hays

**Affiliations:** 1 Medicine, Johns Hopkins, Baltimore, MD, USA; 2 University of PIttsburgh, Pittsburgh, PA, USA; 3 German Heart Institute, Berlin, Germany

## Background

We previously reported that coronary distensibility can be measured non-invasively using 3T MRI and that distensibility is reduced in coronary artery disease (CAD) patients compared with healthy subjects. Because atherosclerotic vascular disease is an inflammatory process, we sought to test the hypothesis that coronary distensibility measured with MRI is associated with systemic inflammation, measured by serum high sensitivity C reactive protein, hsCRP, a marker of increased inflammation, and adiponectin, a fat hormone associated with decreased inflammation, in healthy subjects and in CAD patients.

## Methods

25 subjects (8 healthy adults, age=38.6±6.5years,mean±standard error) and 17 patients with catheterization-documented CAD (age=59.4±3.4) were studied using a commercial whole-body MR imaging system (Achieva 3.0T;Philips,Best,Netherlands). Spiral cine MRI was performed for area measurements of a proximal coronary artery. MRI parameters were:echo time (TE)=1.5ms,radiofrequency(RF) excitation angle=20°;spectral spatial excitation,acq. window=10ms,repetition time(TR)=14ms,21 spiral interleaves,spatial resolution(acquired/reconstructed)=0.89x0.89x8.00mm^3^/0.69x0.69x8.00mm^3^. Blood pressure and heart rate were recorded. Images were analyzed for cross-sectional area in systole and diastole using semi-automated software (Cine vs3.15.17,General Electric), and distensibility(mmHg^-1^) was determined as: [(systolic lumen area-diastolic lumen area)]/(pulse pressure multiplied by diastolic lumen area). Blood was obtained the same day as the MRI to quantify serum hsCRP and adiponectin (in a subset,n=16).

## Results

**Figure 1:**
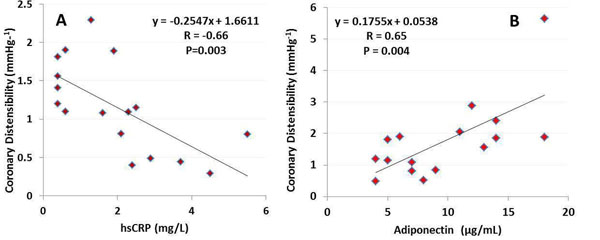
A: Coronary artery distensibility (mmHg^-1^) is inversely related to serum hsCRP (high sensitivity C reactive protein) level in patients with coronary artery disease (CAD, N=17). B: Adiponectiv (a fat hormone that suppresses inflammation) is positively related to coronary distensibility in CAD patients and healthy adults combined (N=16).

Coronary distensibility was impaired in the CAD group, (1.16±0.17mmHg^-1^) as compared to that in the healthy subjects (2.17±0.57 mmHg^-1^p=0.05). Mean hsCRP was higher in the CAD group, 2.0±0.49mg/L, but did not differ significantly from that in the healthy group (1.47±0.48mg/L(range 0.4-5.5,p=NS) and mean adiponectin levels also did not differ between the CAD (8.1±1.71µg/mL) and healthy (11.2±1.52µg/mL,p=NS) groups. Although there was no relationship between hsCRP and coronary distensibility in healthy subjects (R=0.36, p=0.37), there was a strong inverse relationship between CRP and distensibility in CAD patients (R=-0.66,p=0.003, Figure 1A) and a positive relationship between adiponectin level and coronary distensibility in both the healthy and in the CAD subjects and the combined group(R=0.65, p=0.004,Figure 1B).

## Conclusions

Coronary distensibility measured noninvasively by 3T MRI is impaired in CAD patients compared to healthy controls and the degree of distensibility in CAD patients is inversely related to hSCRP level, a marker of inflammation. Adiponectin, a fat hormone known to suppress inflammation, is positively related to distensibility. These data suggest that systemic inflammation is closely related to coronary distensibility. Further studies are needed to determine whether suppressing inflammation improves coronary distensibility and other measures of vascular disease.

## Funding

NIH/NHLBI grants R01HL084186, AHA SDG 5200004, AHA 12PRE11510006.

